# Alterations in gut microbiota improve SCFA production and fiber utilization in Tibetan pigs fed alfalfa diet

**DOI:** 10.3389/fmicb.2022.969524

**Published:** 2022-10-21

**Authors:** Qingtao Gao, Guangming Sun, Jiujun Duan, Chengzeng Luo, Cidan Yangji, Ruqing Zhong, Liang Chen, Yanbin Zhu, Basang Wangdui, Hongfu Zhang

**Affiliations:** ^1^Institute of Animal Husbandry and Veterinary Medicine, Tibet Academy of Agriculture and Animal Husbandry Science, Lhasa, China; ^2^The State Key Laboratory of Animal Nutrition, Institute of Animal Sciences, Chinese Academy of Agricultural Sciences, Beijing, China; ^3^College of Animal Science, Xinjiang Agricultural University, Urumqi, China

**Keywords:** alfalfa, Tibetan pigs, microbiota, SCFA, *in vitro* fermentation

## Abstract

Tibetan pigs were thought to have good performances of rough feeding tolerance, which may be related to the gut microbiota. This study was conducted to investigate the changes of colonic microbiota contribute to fiber utilization in Tibetan pigs fed alfalfa supplementation diet compared with basal diet, and verified whether the microbial community in Tibetan pigs fed alfalfa diet was beneficial to utilize fiber using *in vitro* fermentation. A total of 40 Tibetan pigs were allocated into two groups and fed with a corn-soybean meal basal diet (CD) or a 50% alfalfa supplementation diet (AD) for 42d. Our results showed pigs fed CD diet improved carcass weight compared to pigs fed AD diet (*p* < 0.05), yet reduced the bacterial diversity (*p* < 0.05). Tibetan pigs fed CD diet increased certain pathogenic bacteria (*Streptococcus*) abundance (FDR < 0.05). Alfalfa consumption increased fiber-degrading bacteria abundance (*UCG-005, Rikenellaceae_RC9_gut_group, Prevotellaceae_UCG-003, Alloprevotella, Marvinbryantia,* and *Anaerovibrio*) in the colonic digesta (FDR < 0.05) and improved concentrations of acetate, propionate, butyrate, and total SCFA in colonic content (*p* < 0.05). Higher fermentation capacity of fecal microbiota from pig fed AD diet was verified by *in vitro* fermentation. Collectively, our results indicated that alfalfa supplementation in diets improved the abundance of fiber-degrading bacteria and SCFA production in the hindgut of Tibetan pig, as well as enhanced the fermentation capacity of fecal microbiota.

## Introduction

Fiber-rich ingredients are abundant and inexpensive feed sources, which can be used to partially replace corn and soybean meal (SBM) in diets. Studies have reported that adding appropriate levels of fibrous ingredient to diet could effectively reduce the costs and maintain animal health (Koh et al., 2016; Li et al., 2021b), by preventing metabolic disorders and improving gut health (Williams et al., 2001; Jha et al., 2019; Gill et al., 2021). Whereas, pigs had been reported to have poor ability to utilize fiber fractions (Urriola et al., 2010). Furthermore, the addition of fibers to diet can adversely affect the utilization of other nutrients (Chen et al., 2013). Several strategies, such as feed-processing techniques and use of fiber-degrading enzymes, have been employed to ameliorate the negative effects associated with the ingestion of high-fiber diets in pigs (Agyekum and Nyachoti, 2017). Microorganisms, including fungi and bacteria, are the common sources of cellulases (Ega et al., 2020). Recently, isolation, screening, and identification of specialized bacterial cellulose degraders from the animal guts have aroused much attention, which provided a novel perspective for improving the nutritive value of high-fiber diets in pigs (Yang et al., 2014; Zhou et al., 2020).

Chinese indigenous pig breeds are known for their adaptability to extensive feeding and good performance in crude fiber utilization (Jiang et al., 2011). Tibetan pigs, raised mostly on alfalfa pasture or fed roughage, are local domesticated breed of the Qinghai-Tibet Plateau and has the characteristics of rough feeding tolerance and strong disease resistance (Yang et al., 2011). Efficient digestion of dietary fiber has been reported to be associated with the gut microbiota in pigs (Jha and Berrocoso, 2015; Cheng et al., 2018). The Tibetan pig’s herbivorous nature may also be closely linked to its unique gut microbial composition. According to a study, cellulolytic bacterium (*Bacillus subtilis* BY-2) was isolated and identified from the Tibetan pig’s intestine and cellulase production by this bacterium was further investigated (Yang et al., 2014). Cellulolytic bacteria can be added directly to diet and can also be utilized to produce cellulase for application. It is important to explore more fibrolytic-degrading bacteria, because the diversity of microorganisms can adapt to the varied fibrous ingredients. Other study has reported that grazing Tibetan pigs had higher bacterial diversity and higher digestibility of fiber components compared to captured Tibetan and DLY pigs. However, under the same feeding conditions, the cecal microbial community diversity and the digestibility of fiber components of captured Tibetan pigs and DLY pigs were similar (Tan, 2021). This indicated that diet is a significant factor in shaping gut microbiota composition and suggested that the rough feeding resistance of Tibetan pigs may be largely ascribed to stronger adaptability to dietary fiber of the gut microbiota.

Diet with different fiber levels may differently affect gut microbiota, leading to differently adapted bacterial communities. Alfalfa is a high-quality fiber feedstuff and is mainly composed of IDF (Chen et al., 2013; Wang et al., 2018), which also containing an abundance of minerals and vitamins, as well as moderately rich in protein, well balanced in amino acids (Jiang et al., 2012). Alfalfa-containing diets could alter luminal microbiota structure of pigs (Wang et al., 2018). However, the adaptation of Tibetan pig intestinal microbiota composition after being fed low fiber diet or high level of alfalfa supplementation diet remains unclear, and whether the ingestion of alfalfa could remain the fiber utilization capacity of gut microbiota still needs further verification. We tried to analyze the underlying physical mechanisms of efficient fiber utilization in Tibetan pigs from the perspective of gut microbiota. We hypothesized that the microbiota of Tibetan pigs fed commercial diets would reduce the ability of readily utilize fiber components, whereas, after dietary alfalfa supplementation, Tibetan pig gut microbiota could remain the property of the rough feeding tolerance and be suitable for degrading fiber components. Therefore, the objective of this study was to investigate the alterations of gut microbiota composition and short-chain fatty acids (SCFA) profiles to alfalfa supplementation, and verified the role of intestinal microbes in the crude feeding tolerance of Tibetan pigs by *in vitro* fermentation technique.

## Materials and methods

### Animals, management, diets, and experimental design

**Figure 1:**
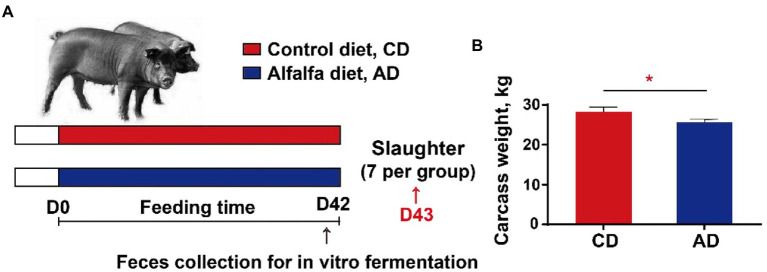
Experimental design **(A)** and carcass weight **(B)**. *p < 0.05. CD, control diet; AD, alfalfa diet.

A total of 40 Tibetan pigs (23.6 ± 2.2 kg) with the same genetic background and feeding management condition were allocated into two groups and fed twice daily with a corn-soybean basal diet (CD) or a 50% alfalfa supplementation diet (AD), respectively (Table 1). All pigs were allowed free access to feed and water. Diets were formulated to meet or exceed the vitamins and minerals of pigs according to the nutrient requirements of swine (GB/T 39235–2020). At d42, feces of 5 pigs in each group were individually collected into airtight bags immediately after defecation, expelling air to maintain anaerobic conditions, then stored at −80°C. After 42-d feeding trial, 7 pigs were randomly selected from each group and euthanized (Figure 1A). The carcass weight was recorded immediately. And the colonic digesta from each group were sampled, which were immediately frozen in liquid nitrogen, and then stored at −80°C for microbial 16S rDNA sequencing and SCFA analysis.

**Table 1:** Composition and nutrient level of experimental diet (as fed-basis).

Ingredients	Corn diet	Alfalfa diet
Corn	66.4	32.14
Soybean meal	15.5	7.50
Wheat bran	15	7.26
Alfalfa	0	50
Limestone	1.00	1.00
CaHPO4	0.5	0.5
NaCl	0.3	0.3
L-Lys (70%)	0.2	0.2
Choline chloride	0.1	0.1
Premix^1^	1	1
Total	100.00	100.00
Calculated nutrient levels, %		
CP	14.83	15.37
DE, MJ/kg	13.90	9.77
NE, MJ/kg	10.37	7.08
SID Thr	0.43	0.43
SID Trp	0.13	0.12
SID Lys	0.75	0.64
SID Met	0.23	0.20
Ca	0.56	1.09
TP	0.46	0.42
STTD P	0.25	0.25

1 Provided the following quantities per kg of diet: vitamin A, 9,140 IU; vitamin D_3_, 4,405 IU; vitamin E, 11 IU; menadione sodium bisulfite, 7.30 mg; riboflavin, 9.15 mg; D-pantothenic acid, 18.33 mg; niacin, 73.50 mg; choline chloride, 1,285 mg; vitamin B_12_, 200 ug; biotin, 900 ug; thiamine mononitrate, 3.67 mg; folic acid, 1,650 ug; pyridoxine hydrochloride, 5.50 mg; I, 1.85 mg; Mn, 110.10 mg; Cu, 7.40 mg; Fe, 73.50 mg; Zn, 73.50 mg; Se, 500 ug.

### DNA extraction, 16S rDNA sequencing, and data processing

The total DNA of colonic digesta samples was extracted with Qiagen DNA isolation kit (Qiagen, Hilden, Germany) according to the protocol, the quality and quantity of DNA were detected (Supplementary Figure S1A). PCR was performed in an ABI GeneAmp^®^ 9700 thermocycler (ABI, CA, United States) targeting the V3–V4 hypervariable regions of the 16S rDNA with primers 338F (5’-ACTCCTRCGGGAGGCAGCAG-30) and 806R (50-GGACTACCVGGGTATCTAAT-3′). The PCR reaction conditions were performed as follows: 95°C for 3 min; 27 cycles of 95°C for 30 s, 55°C for 30 s, and 72°C for 45 s; and elongation step at 72°C for 10 min, then end at 4°C. PCR products were detected using a 2% (w/v) agarose gel (Supplementary Figure S1B) and purified with AxyPrep DNA Gel (Axygen). The sequencing of amplicons was conducted on the Illumina HiSeq sequencing platform by Majorbio Bio-Pharm Technology Co. Ltd. (Shanghai, China). The clustering of clean sequences into operational taxonomic units (OTUs) was performed using UPARSE (version 7.0.1090)^1^ at the 97% threshold (Edgar, 2013). OTUs were performed against the Ribosomal Database Project classifier (RDP, version 11.1)^2^ at a confidence threshold of 0.7 (Wang et al., 2007).

### Quantification of short-chain fatty acids

The concentration of SCFAs in the colonic digesta was determined using gas chromatography according to previously described with slight modifications (Tang et al., 2021). In brief, about 0.5 g wet digesta was mixed with ultrapure water for 12 h, and centrifugation at 10,000 ×*g* for 10 min at 4°C to obtain the supernatant. Metaphosphoric acid (25%, w/v) was mixed with the extracts at a ratio of 2:8. After centrifugation at 12,000 ×*g* for 15 min at 4°C, the supernatant was passed through the 0.45-μm Milled-LG filter (Millipore, Billerica, MA, United States) and subjected for SCFA analysis.

### Comparation of fiber-degrading ability of fecal microbiota

*In vitro* fermentation was performed to verify the difference in fiber utilization ability between fecal microbiota of pigs fed CD or AD diet. CD diet (SCD), AD diet (SAD), and alfalfa ingredient (SAI) were used as substrates. Inocula were prepared according to previously reported procedures (Gao et al., 2022). In brief, feces collected from pigs in AD or CD group were equally mixed and diluted with pre-warmed buffer solution at ratio of 1:5 (w/v), respectively, then stirred and filtered, which served as inocula. The medium was a buffer solution composed of salts and minerals, which was modified as our previous study (Gao et al., 2022).

**Figure 2:**
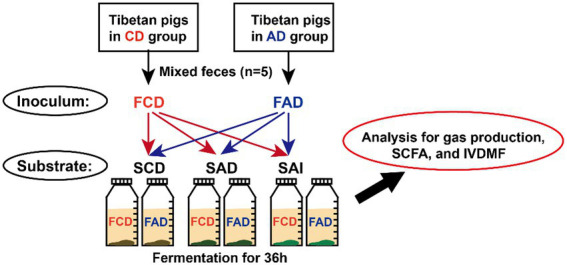
Schematic diagram representing the *in vitro* fermentation designs. FCD, feces of CD group; FAD, feces of AD group; SCD, substrate of control diet; SAD, substrate of alfalfa diet; SAI, substrate of alfalfa ingredient.

Three substrates were fermented using the two inocula with 5 replicates for each treatment (Figure 2). Blank glass bottles without substrates were used as controls (5 replicates for each inoculum). Substrates were weighted at 0.5 g into 100 ml fermentation bottles, then added with 60 ml medium and 5 ml inoculum. All bottles were sealed and incubated for 36 h at 39°C in a temperature-controlled incubator. All the procedures were operated under anaerobic conditions in an anaerobic chamber.

The gas production of each bottle was repeatedly recorded at 12, 14, 16, 18, 20, 24, and 36 h with an air pressure transducer (SUAY30, RFS). The gas production recordings were fitted to a non-linear model according to Groot et al. (1996) using the JMP 10.0 software (SAS Institute, Cary, NC, United States):

$${\mathrm{GP}}_{t}=A/\left(1+{\left(C/t\right)}^{B}\right)$$

AGPR=(A×B)/(4×C)

Where GP_t_ (mL/g) is the total gas production; A (mL/g), the maximum gas volume; C (h), the time to half asymptote; AGPR (mL/g h^−1^), the gas production rate when the gas production reaches 1/2 of the maximum gas production.

After 36 h fermentation, bottles were placed in the ice-water to stop fermentation. Fermentation broth was centrifuged (10,000 ×*g*, 10 min, 4°C), two milliliters of supernatant were sampled for SCFAs (Acetate, Propionate, and Butyrate) analysis. All other supernatant and precipitate were collected by filtration (40 μm filter paper, Whatman 1,541), washed with ethanol (20 ml, 95%), and acetone (20 ml, 99.5%); dried for 4 h at 105°C, and weighed for determination of IVDMF:

$$\mathrm{IVDMF}\;\left(\%\right)=\left(1-\left(M_{2}-M_{3}\right)/M_{1}\right)\times 100\%$$

Where IVDMF, *in vitro* dry matter fermentability; M_1_, the weight of substrate (g, DM basis), M_2_, the weight of residues after substrates fermentation (g, DM basis); M_3_, the weight of residues after blank controls fermentation (g, DM basis).

### Statistical analysis

With the pig as the experimental unit, student T test was conducted to compare the carcass weight and SCFA concentration in colonic digesta using SAS 8.0 (SAS Institute, Inc., Cary, NC, United States). For the alpha diversity (Sobs, Shannon, ACE, and Chao indexes) and beta diversity, student’s t-test or the principal coordinate (PCoA) analysis based on the Bray-Curtis distance and ANOSIM test were performed using Majorbio Cloud Platform^3^ according to the relative abundance of OTUs. The significant difference between diet treatments at the genus level was tested by Wilcoxon rank-sum test with corrected *p*-value (FDR) < 0.05. Moreover, Redundancy analysis (RDA) and Spearman’s or Mantel’s correlation analysis were performed using the R vegan and ggcor packages.

The data of *in vitro* fermentation experiment including fitted gas production parameters (A, B, AGPR), IVDMF, and SCFA production in fermentation broth were analyzed using Proc GLM by SAS with each fermentation bottle as the experimental unit. And the procedure included two fixed factors (substrate and inoculum) as well as their interaction. To further compared the difference between inoculum within substrate, a two-sample comparison was also employed using the TTEST procedure of SAS. *p* < 0.05 and 0.05 < *p* < 0.1 were considered as statistically significant and significant trend, respectively.

## Results

### Carcass weight

Tibetan pigs in two groups had comparable initial body weight. After 42d feeding experiment, pigs in AD group had lower carcass weight compared with pigs in CD group (28.29 ± 0.31 kg vs. 25.64 ± 0.44 kg, *p* < 0.05; Figure 1B).

### Estimation of sequencing data

After merging and filtering, an overall number of 452,623 valid sequences, including 217,545 sequences for CD group and 235,078 sequences for AD group, from 14 colon digesta samples were retrieved with ranged from 27,698 to 35,729 per sample. The clean data were normalized into the depth of 27,698 reads in each sample, based on the minimum number of sample sequences, and clustered into 1,400 OTUs at a 97% similarity. All of 1,400 OTUs were clustered into 240 genera, 95 families, 57 orders, 27 classes, and 16 phyla. Good coverage (>99.4%) and rarefaction curves of Sobs and Shannon indicated the sequencing depth is sufficient (Supplementary Figure S2A, S1B). Rank-Abundance curves reflected good richness and evenness of bacterial species in samples in each group (Supplementary Figure S2C). Moreover, Venn diagram exhibiting the overlaps between two groups presented that 938 OUTs of the total richness of 1,400 OTUs were shared, 170 OTUs were unique to the CD group, and 292 OTUs were unique to the AD group (Supplementary Figure S2D).

### Gut microbial diversity

**Figure 3:**
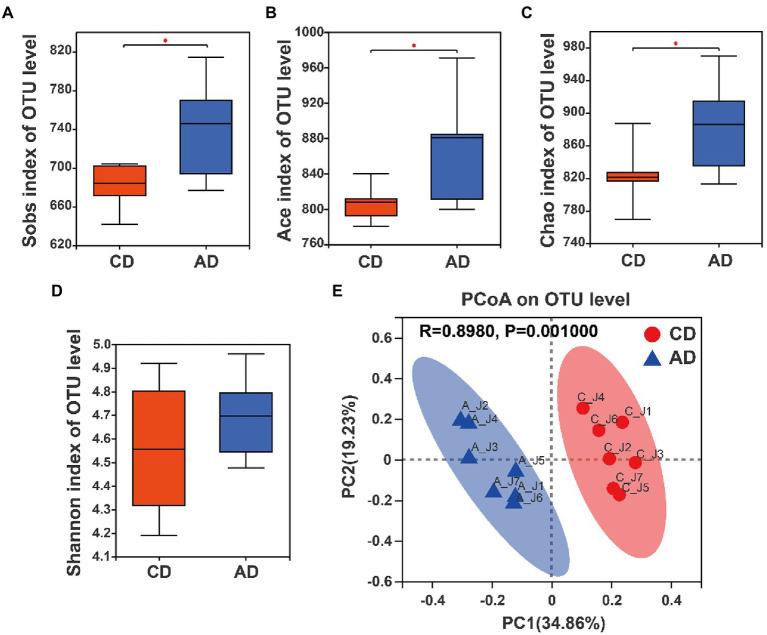
Alpha-diversity analysis and beta-diversity of microbiota composition in colonic samples among CD and AD groups. **(A)** Sobs index, **(B)** Ace index, **(C)** Chao index, **(D)** Shannon index. **(E)** PCoA analysis based on Bray–Curtis distance matrix with ANOSIM test. ^*^Indicates *p* < 0.05 between CD and AD group. CD, control diet; AD, alfalfa diet.

Microbial α-diversity analysis showed that no significant difference in Shannon index was observed between pigs in CD and AD group (Figure 3D), whereas there were higher Sobs index, Ace index, and Chao index of microbial community in colonic digesta of pigs in AD group compared with those of pigs in CD group (*p* < 0.05; Figures 3A–C). Furthermore, β-diversity analysis based on the Bray-Curtis distance and ANOSIM test was performed, and the results revealed that community structure of microbiota formed significant clustering (*R* = 0.898, *p* = 0.001; Figure 3E) between CD and AD groups. Collectively, these results indicated a significant improvement in gut microbial diversity in the AD group.

### Microbial community in phylum and genus level

On the phylum level, a total of 16 bacterial phyla were detected in colonic digesta samples. In which, Firmicutes (76.57% for AD group, 83.45% for CD group) and Bacteroideta (17.35% for AD group, 11.46% for CD group) were 2 dominant bacterial phyla, followed by Spirochaetota and Actinobacteriota, accounting for 97.35 ~ 99.15% of the total bacterial valid sequences (Supplementary Figure S3A). Dietary alfalfa supplementation had the tendency that increased Bacteroidota abundance (*p* = 0.055; Supplementary Figure S3B) and decreased the relative abundance ratio of Firmicutes/Bacteroidota (*p* = 0.096; Supplementary Figure S3C).

**Figure 4:**
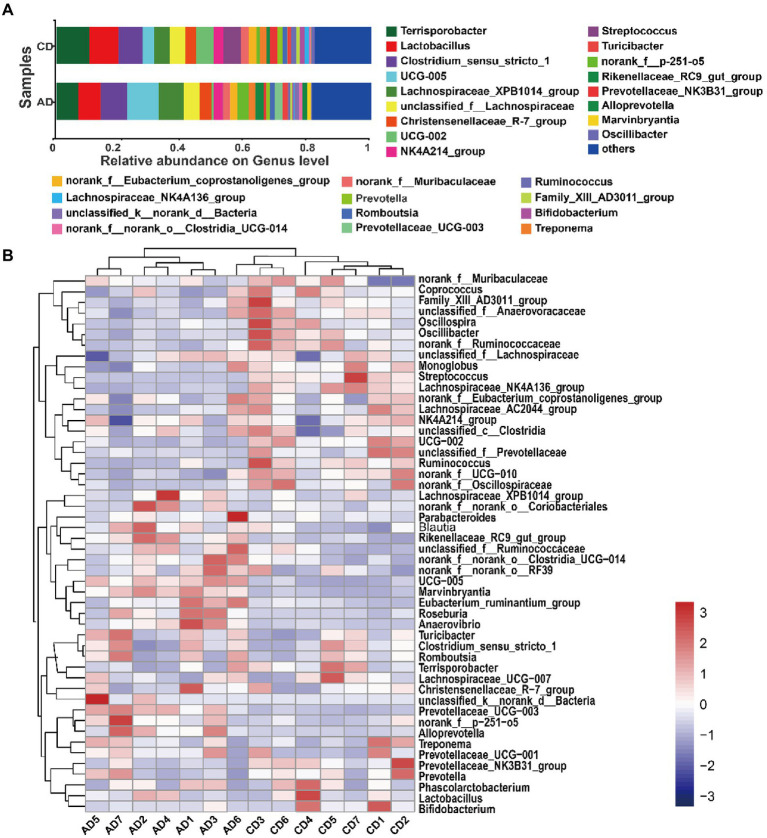
Microbial community. Microbiota composition of colonic digesta and relative abundance (>0.01) at genus levels **(A)**. Hierarchically clustered heatmap analysis of microbial community (top 50) among 14 samples at the genus level **(B)**. CD, control diet; AD, alfalfa diet.

On the Genus level, there were 29 genera with relative abundances of over 1.0% in at least one group, accounting for 81.14 ~ 81.92% of the total bacterial valid sequence. The dominant genera (top 10) observed included *Terrisporobacter*, *Lactobacillus*, *Clostridium_sensu_stricto_1*, *UCG-005*, *Lachnospiraceae_XPB1014_group*, *unclassified_f__Lachnospiraceae*, *Christensenellaceae_R-7_group*, *UCG-002*, *NK4A214_group*, and *Streptococcus*, consisting of more than 52% of total genera (Figure 4A). Unsupervised hierarchical clustering was performed based on the similarity of relative abundance of microbial genera, and showed that samples were clustered into 2 main groups (Figure 4B), indicating a more similarity of the samples within group than between groups.

### Alterations of microbiota composition

**Figure 5:**
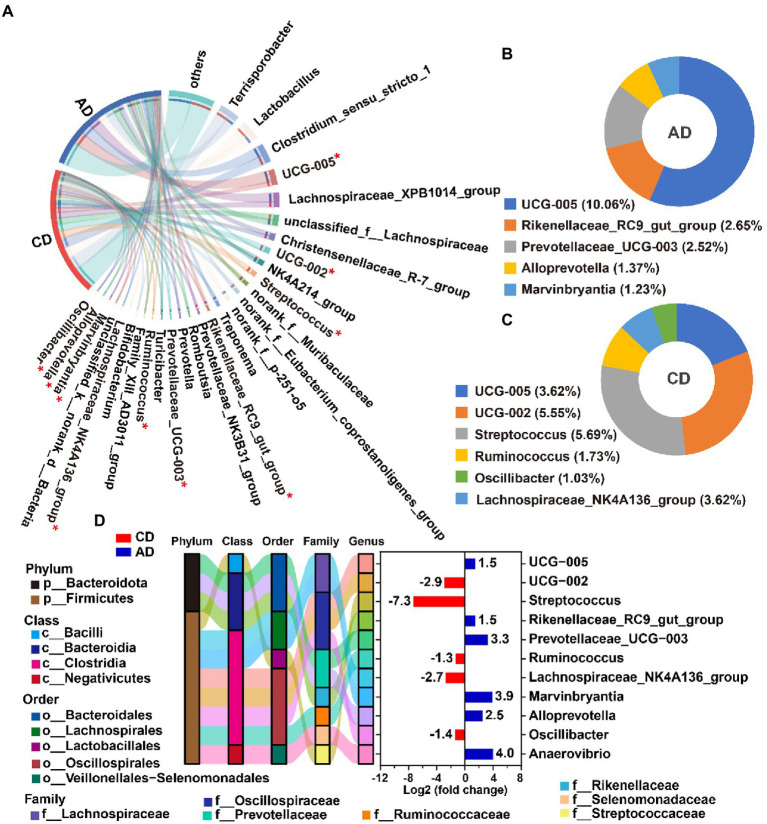
Differential genera analysis between CD and AD groups. The top 1% genera (including differentially abundance genera with FDR <0.05) from colon digesta of pigs in CD and AD group. **(A)** The enriched differential genera with relative abundance over 1% in AD group **(B)** and CD group **(C)**; Taxonomy and differentially abundant genera (in the relative abundance of top 50) from colon digesta of pigs in AD group compared to CD group **(D)**. Positive values indicate that genera enriched in the AD group, while negative values suggest that genera enriched in the CD group. ^*^Indicates FDR <0.05. CD, control diet; AD, alfalfa diet.

Kruskal-Wallis H test was employed to identify the specific genus in the colonic microbiome that was notably associated with alfalfa supplementation. A total of 11 microbiotas (top 50), included 5 downregulated genera (*g__UCG-002*, *g__Streptococcus*, *g__Ruminococcus*, *g__Lachnospiraceae_NK4A136_group*, and *g__Oscillibacter*) and 6 upregulated genera (*g__UCG-005*, *g__Rikenellaceae_RC9_gut_group*, *g__Prevotellaceae_UCG-003*, *g__Alloprevotella*, *g__Marvinbryantia*, and *g__Anaerovibrio*) were detected (FDR < 0.05, Figures 5A,D). All the notably different genera were classified into Firmicutes and Bacteroidota (Figure 5D). The differential genera with relative abundance over 1% in AD group were *g__UCG-005*, *g__UCG-002*, *g__Streptococcus*, *g__Ruminococcus*, *g__Lachnospiraceae_NK4A136_group*, and *g__Oscillibacter* (Figure 5B), and those in CD group were *g__UCG-005*, *g__Rikenellaceae_RC9_gut_group*, *g__Prevotellaceae_UCG-003*, *g__Alloprevotella*, and *g__Marvinbryantia* (Figure 5C). Among the differential microbiota, genera *Streptococcus* was enriched in CD group and 156-fold higher than AD group (Figure 5D).

### Comparation of SCFA concentration

**Figure 6:**
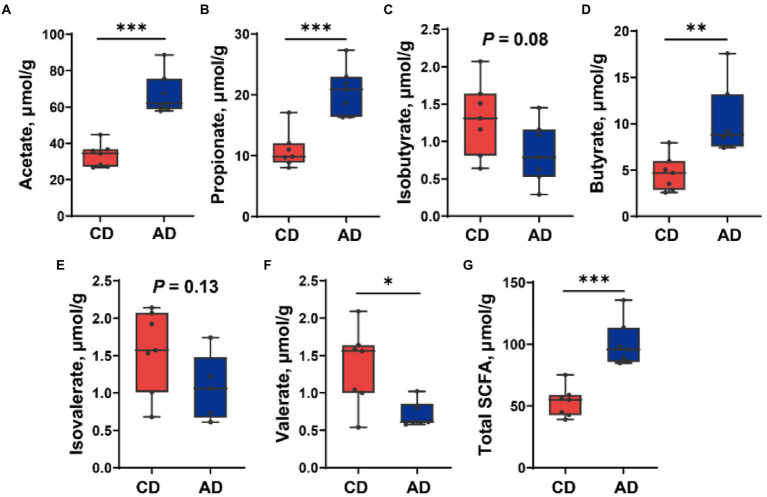
Short-chain fatty acids (SCFAs) concentration in colonic digesta of pig in CD and AD groups. Concentration of **(A)** Acetate, **(B)** Propionate, **(C)** Isobutyrate, **(D)** Butyrate, **(E)** Isovalerate, **(F)** Valerate, and **(G)** Total SCFA. ^*^p < 0.05, ^**^p < 0.01 and ^***^p < 0.001. CD, control diet; AD, alfalfa diet.

Further investigation of SCFA concentration in colonic digesta of pigs fed different diet was completed (Figure 6). Dietary alfalfa supplementation improved the concentration of acetate, propionate, butyrate, and total SCFA (*p* < 0.01), diminished valerate yield (*p* < 0.05), and tended to decrease isobutyrate level (*p* = 0.08).

### Microbiota-SCFAs correlation

**Figure 7:**
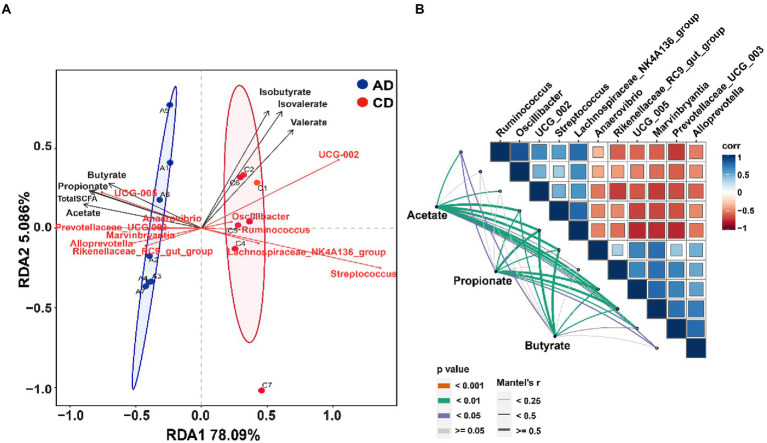
Correlation analysis of microbiota and SCFA. **(A)** Redundancy analysis of the colonic microbiota composition at genus level relative to colonic SCFAs; **(B)** Pairwise comparisons of different genera are demonstrated with a color gradient denoting Spearman’s correlation coefficient. Acetate, propionate, and butyrate are related to each microbial genus by Mantel’s correlation analysis. CD, control diet; AD, alfalfa diet.

RDA analysis was completed and shown in Figure 7A. The results indicated that the microbiota composition of colonic samples in the CD group was clearly separated from those in the AD group at the first constrained axis. Genera enriched in AD group were positively correlated with acetate, propionate, butyrate, and total SCFA, while genera enriched in CD group were positively correlated with isobutyrate, isovalerate, and valerate. To further explore the association between colonic bacteria and SCFAs, Spearman or Mantel correlation analyzes were conducted by the abundance of differential colonic genera and concentration of SCFAs (acetate, propionate, and butyrate). The results demonstrated that most genera (*g__UCG-002*, *g__Streptococcus*, *g__Ruminococcus*, *g__Lachnospiraceae_NK4A136_group*, and *g__Oscillibacter*) belonged to Firmicutes were negatively correlated with genera (*g__Rikenellaceae_RC9_gut_group*, *g__Prevotellaceae_UCG-003*, and *g__Alloprevotella*) belonged to Bacteroidota. Excepting genus *g__Oscillibacter*, all other genera were significantly associated with acetate (Figure 7B, Mantel’s *r* > 0.25, *p* < 0.05). In addition, there were significant correlations between seven genera (*g__Ruminococcus*, *g__Streptococcus*, *g__Lachnospiraceae_NK4A136_group*, *g__Anaerovibrio*, *g__UCG-005*, *g__Marvinbryantia*, and *g__Prevotellaceae_UCG-003*) and butyrate production (Figure 7B, Mantel’s *r* > 0.25, *p* < 0.05). Besides the same 7 genera, another genus *g__Alloprevotella* was also significantly related to propionate production (Mantel’s *r* > 0.25, *p* < 0.05).

### Gas production and IVDMF during *in vitro* fermentation

The gas production curves and fitted parameters were depicted in Supplementary Figure S4; Table 2. The results showed that significant differences in fermentation capacity of fecal microbiota from AD or CD groups were observed. There were significantly higher A (mL/g), AGPR (mL/g h^−1^), and lower **C** (h) during *in-vitro* fermentation using feces from AD group in comparison to feces from CD group (*p* < 0.01; Table 2), whichever substrate was fermented. Meanwhile, the fermentation performance varies significantly among substrates (*p* < 0.01; Table 2; Supplementary Figure S4A), in which control diet fermentation displayed the fastest kinetics, followed by alfalfa supplementation diet, and alfalfa ingredient showed the slowest kinetics (*p* < 0.01; Table 2). Moreover, significantly higher IVDMF was obtained during *in-vitro* fermentation using feces from AD group compared with feces from CD group (*p* < 0.01; Table 2), regardless of substrate. And control diet was found the highest IVDMF, followed by alfalfa diet and then alfalfa ingredient (*p* < 0.05).

**Table 2:** Gas fermentation parameters (A, C, AGPR) and IVDMF of *in vitro* fermentation using inoculum prepared by feces from pigs fed CD or AD diet.

Substrates	Inoculum	A (mL/g)	C (h)	AGPR (mL/g h^−1^)	IVDMF, %
SCD	FAD	272.7^a^	13.3^b^	23.8^a^	76.7^a^
	FCD	250.8^b^	15.9^a^	16.1^b^	73.8^b^
SAD	FAD	188.4^a^	13.5^b^	12.8^a^	57.1^a^
	FCD	169.3^b^	16.1^a^	8.7^b^	52.3^b^
SAI	FAD	125.2	15.5^b^	7.9^a^	42.1^a^
	FCD	108.5	18.6^a^	3.3^b^	36.8^b^
SEM		3.24	0.24	0.44	0.81
*P*-value					
Inoculum		<0.01	<0.01	<0.01	<0.01
Substrate		<0.01	<0.01	<0.01	<0.01
I*S		0.73	0.53	<0.01	0.31

For each substrate, different superscripts (a, b) indicate a significant difference (*p* < 0.05). A, the maximum gas volume; C, the time to half asymptote; AGPR, the gas production rate when the gas production volume reaches 1/2 of the maximum gas production volume; IVDMF, *in vitro* dry matter fermentability. FCD, feces of CD group; FAD, feces of AD group; SCD, substrate of control diet; SAD, substrate of alfalfa diet; SAI, substrate of alfalfa ingredient.

### SCFA production during fermentation

**Figure 8:**
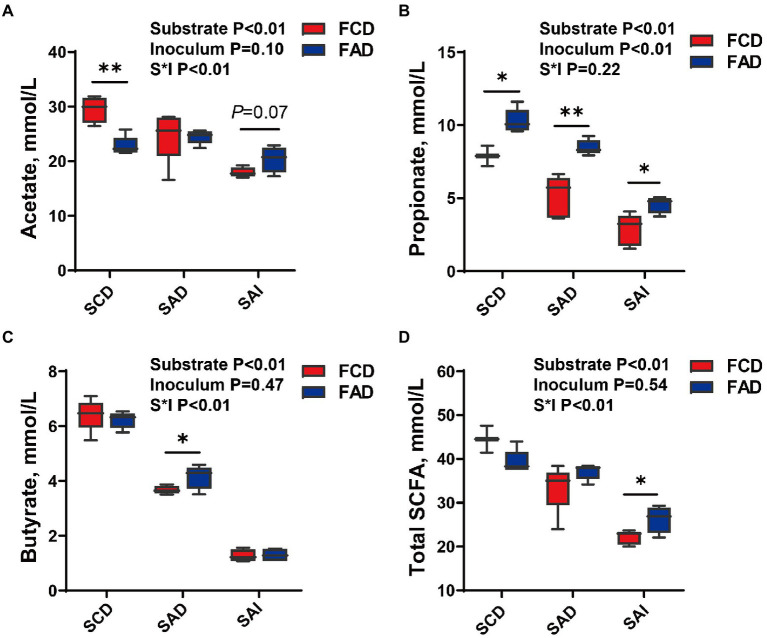
SCFA production during *in-vitro* fermentation of different substrate using feces from pigs fed different diets. Concentration of **(A)** Acetate, **(B)** Propionate, **(C)** Butyrate, and **(D)** Total SCFA. ^*^p < 0.05, ^**^p < 0.01. FCD, feces of CD group; FAD, feces of AD group; SCD, substrate of control diet; SAD, substrate of alfalfa diet; SAI, substrate of alfalfa ingredient.

The fermentation of feces from AD group produced higher propionate than feces from CD group (*p* < 0.05; Figure 8B), irrespective of substrate. The production of acetate, butyrate, and total SCFA showed a substrate dependence, in which significant interaction effects of substrates and inoculum were found (*p* < 0.05; Figures 8B–D). The acetate production of SCD fermentation was greater using feces from CD group than feces from AD group (*p* < 0.05; Figure 8A). With the increased fiber levels of substrate, the production of acetate during fermentation was higher in feces from AD group than CD group (*p* = 0.07; Figure 8A). The propionate production of SAD fermentation was greater using feces from AD group than feces from CD group (*p* < 0.05; Figure 8C). No significant difference was observed for SCD and SAI during fermentation of feces from AD or CD group. Regarding total SCFA production, stronger fermentation ability for feces from AD group was observed with an increment of fiber levels in substrate (*p* < 0.05; Figure 8D). On the other hand, as fiber content increased, the production of total SCFA for feces fermentation from either AD group or CD group gradually decreased (*p* < 0.01).

## Discussion

Tibetan pigs, as an excellent native pig breed, have good performances of strong disease resistance, rough feeding tolerance, and good meat quality (Yang et al., 2011; Gan et al., 2019). Currently, Tibetan pigs fed mostly under traditional free-range conditions or fed with roughage and green feed, which include high dietary fibers content and low energy density. The present study found that Tibetan pigs fed CD diet had higher carcass weight compared with pigs fed AD diet. Poor growth performance of Tibetan pigs may be related to low energy intake. Previously study has reported that Tibetan pigs fed corn-SBM diet had better growth performance than Tibetan pigs raised in grazing, even after they fed the same diet (Tan, 2021). Our results suggested that sufficient nutrient supply is essential for efficient Tibetan pig production. However, whether rapid growth of Tibetan pigs will have a negative effect on meat quality, even on animal health, requires further study. Furthermore, study reported that alfalfa meal-contained diets did not affect the growth performance (ADG, ADFI, and final BW) of commercial pigs (Wang et al., 2018). This may be due to the lower levels of alfalfa supplementation in the previous study, indicating that different graded levels of alfalfa supplementation need to further investigation to explore the balance of feed costs, animal health and growth performance.

Previous study reported that grazing-raised Tibetan pigs had a better ability to digest fiber (Tan, 2021). The differences in the ability to utilize dietary fiber for pigs might be related to the differences in gut microbiota composition. Research into the Tibetan pig gut microbiota is of great importance to understand their roles in digestion and metabolism function. In a study of the evolution of intestinal microbiota in Ningxiang pigs, the results proved that diet was the main driving factor for the alterations of intestinal microbiota (Li et al., 2021a). In which, the increased α-diversity of microbiota were observed with increasing fiber levels in diet and different ages. In the PCoA analysis, there were no clear clusters of the same diet between different ages, but significant separation between diet with different fiber levels was found. That was consistent with the present study that Tibetan pigs fed alfalfa containing diet had higher α-diversity, and the microbial community structure of pigs fed alfalfa containing diet was also separated from those fed control diet. Moreover, the characteristics of fecal samples were closer within group than between groups according to the results of hierarchically clustered heatmap analysis. In line with our result, Tan (2021) reported that the cecal and colonic microbial diversity of grazing Tibetan pigs was significantly higher than that of house-feeding Tibetan pigs. In addition, differences in gut microbiota composition also have been reported between different pig breeds (Pajarillo et al., 2014). For example, Fibrobacter was found in greater proportions in the feces of Tibetan pigs than in those of Yorkshire pigs (Xiao et al., 2017). Significant differences microbial community in colon were observed between captured Tibetan pigs and captured DLY pigs fed the same diet from 30 to 150 days. But further research found that the digestibility of fiber components was not different between house-raised Tibetan pigs and DLY pigs. Collectively, those results indicated that the intestinal microbes of Tibetan pigs undergo strong adaptive changes to high-fiber substrates, enabling them to efficiently utilize fiber, while Tibetan pigs fed with low fiber levels diet may lose their character to efficiently utilize fiber although microbiota also had some changed.

In the current study, the dominant bacteria at phyla levels were Firmicutes and Bacteroidota, alfalfa supplementation increased Bacteroidota abundance and decreased Firmicutes/Bacteroidota abundance in colon of pigs. Our results were in line with previous reports that Firmicutes and Bacteroides were revealed to be the two dominant phyla inhabited in the guts of the Tibetan pigs under semi-grazing, which accounted for 74% in Firmicutes and 17.9% in Bacteroides of total sequences (Niu et al., 2022). The Firmicutes/Bacteroides (F/B) was positively correlated with the energy storage capacity (Jumpertz et al., 2011), so the decreasing F/B ratio may be related to low carcass weight of pigs in AD group. In addition, a study reported that gut microbiota participates in Aflatoxin B1 to damage mutton quality, which are related to the increased F/B ratio (Cao et al., 2021). Tibetan pigs fed CD diet had high F/B ratio suggested that feeding a high-concentrate diet to Tibetan pigs for a long-term may result in the depression of meat quality. The dominant genera observed in the current study included *Terrisporobacter*, *Lactobacillus*, *Clostridium_sensu_stricto_1*, *UCG-005*, *Lachnospiraceae_XPB1014_group*, which is consistent with previous results of dominant bacteria of Tibetan pigs (Niu et al., 2022) or other local pigs (Li et al., 2021a).

The present study showed that compared with pigs fed CD diet, feeding pigs with AD diet led to significantly decreased abundance of *Oscillibacter*. It has been reported that genus *Oscillibacter* was positively related to obesity (Gong et al., 2020), which was consistent with the lower body weight of Tibetan pigs fed AD diet. The decrement of *Streptococcus* abundance in alfalfa diet group indicated that alfalfa supplementation could reduce the abundance of certain pathogenic bacteria in the intestinal tract of Tibetan pigs. In agreement with our results, previous studies have also reported that alfalfa-containing diet significantly decreased the potential pathogen *Streptococcus suis* in the caecum and distal colon of piglets (Zhang et al., 2016). In addition, Wang et al. (2018) found that feeding 15% dietary alfalfa meal led to significantly decreased abundance of genera *Acidiphilium*, *Paracoccus*, *Pseudomonas*, and *Acinetobacter*, which are reported as potentially pathogenic and can cause diseases in human and animal (Shin et al., 2015). The results supported our view that the alfalfa meal inclusion can decrease pathogens, and thus benefit the gut health of Tibetan pigs.

On the other hand, a previous study reported that with increasing fiber levels in diet, the abundance of bacteria that capable of degrading plant-derived fiber or having strong correlation with SCFAs production was also improved (Li et al., 2021a). Our study showed that 50% alfalfa supplementation could improve the number of *UCG-005*, *Rikenellaceae_RC9_gut_group*, *Prevotellaceae_UCG-003*, *Alloprevotella*, *Marvinbryantia*, and *Anaerovibrio*, which were related to alfalfa fiber hydrolysis and SCFAs production. Agreeing with our results, a study has reported that the abundance of *UCG-005*, belonging to Oscillospiraceae, was high in Tibetan pigs under semi-grazing (Niu et al., 2022). As reported in another study, genus *UCG-005* was fiber-degrading bacteria and significantly correlated with acetate and total SCFAs concentration (Li et al., 2021a). Also, it has been reported that *Rikenellaceae_RC9_gut_group* is related to being capable of utilizing fibers and generating SCFAs in the hindgut (Qiu et al., 2019). *Anaerovibrio*, which belongs to the Veillonellaceae family, is known to produce propionate as a major fermentation product (Baldwin et al., 2004). Genus *Marvinbryantia*, which is classified into Clostridium cluster XIVa, was positively correlated with intestinal epithelial cell energy metabolism and butyrate production (Louis et al., 2007; Rajilić-Stojanović and de Vos, 2014). Wang et al. (2018) also found increased *Marvinbryantia* abundance in the hindgut of pigs fed alfalfa meal diet. Those results were in agreement with the high acetate, propionate, butyrate, and total SCFA concentration in the colon of Tibetan pigs fed alfalfa supplementation diet. *Alloprevotella* and *Prevotellaceae_UCG-003* are belonging to the family of *Prevotellaceae*, which have the common characteristics that have the potential to modulate intestinal inflammation (Koh et al., 2016; Ning et al., 2020), and also have the capacity to degrade fiber polysaccharides contained in the alfalfa meal (Varel and Yen, 1997; Wang et al., 2018). In addition, *Alloprevotella* was strongly negatively correlated with lipid accumulation and obesity (Kang et al., 2019; Ning et al., 2020), which may also be related to lower carcass weight of pigs fed AD diet. Study has also found that Tibetan pigs fed diet contained 90% green fodder, had large number of disease resistance and fiber digestion bacteria in the cecum (Yang et al., 2018).

Considering the significant variation in the colonic bacterial community of pigs fed high levels of alfalfa meal, the concentration of SCFAs, an important indicator for assessment of fermentation intension in large intestine, was quantified. Previous study has reported that dietary fiber has a great effect on production and absorption of SCFA (Louis and Flint, 2017). We observed the production of acetate, propionate, butyrate and total SCFA were significantly increased in the colon of pigs fed AD diet. The result was supported by the increase of DF content in diet supplemented with 50% alfalfa. Previous study regarding the effects of alfalfa meal on the SCFA concentrations in feces of growing pigs (Chen et al., 2013) was also consistent with our results. Furthermore, the increase in SCFA production in the current study was consistent with changes in gut microbes (such as increased *UCG-005*, *Rikenellaceae_RC9_gut_group*, *Marvinbryantia*, and *Anaerovibrio*).

The gas production kinetics (A, AGPR), SCFA production, and IVDMF are classic indicators reflecting the rate and extent of fermentation (Huang et al., 2017; Uerlings et al., 2019). In the current study, we verified the high fiber utilization of microbiota of Tibetan pigs fed high alfalfa containing diet using *in vitro* fermentation, and the results showed that higher gas production, IVDMF, and SCFAs production by using feces from pigs fed AD diet as inoculum. Our results proved that the adaptive changes of microbiota were happened to utilize high alfalfa diet. With the increased fiber levels of substrate, stronger fermentation ability for feces from AD group was observed. However, the A and AGPR decreased with increasing fiber level of substrate during *in vitro* fermentation using the same fecal inoculum, indicating that the higher the fiber level is, the lower the fermentation rate and extent will be. This could also be confirmed from the result of IVDMF. Consistently, Huang et al. (2017) reported that IVDMF of bran was lower than that of DDGS (23% vs. 69.6%) during *in vitro* fermentation using the same inoculum, because the NDF content of bran was higher than that of DDGS (79% vs. 33%). Whereas, another study showed that increased production of SCFA by *in vitro* fermentation with the increase of substrate fiber level (NDF 33–44.6%, ADF 21.7–26.8%; Iyayi and Adeola, 2015). It can be concluded that the gut microbes of pigs have limited ability to utilize dietary fiber, and the degree and rate of fermentation may increase with the increase of fiber content at appropriate fiber levels. In the current study, moderate level of dietary fiber in substrate (alfalfa diet) increased microbial butyrate production, while low level (control diet) or higher level (alfalfa ingredient) of fiber in substrates have no effect on butyrate yield, which was consistent with previous study regarding the effects of gradient alfalfa meal supplementation on the SCFA concentrations in cecal and colonic digesta of growing DLY pigs (Wang et al., 2018). The reason may be the limited fermentation capacity of fecal microbiota in both two groups for alfalfa, which mainly contains IDF. Whereas the CD diet may easy to be fermented by fecal microbiota in both two groups.

## Conclusion

In summary, our study showed that alfalfa-containing diet could reduce the abundance of certain pathogenic bacteria and improve the abundance of bacteria related to fiber fractions degradation and SCFAs production. High fermentation capacity of fecal microbiota in Tibetan pigs fed alfalfa diet was confirmed using *in vitro* fermentation technique. In future, whether and how diet changed affects disease resistance and meat quality of Tibetan pigs, as well as how to balance the feed cost, growth efficiency, and animal health requires further systemic study.

## Data availability statement

The data presented in the study are deposited in the NCBI Sequence Read Archive database, accession number PRJNA886620.

## Ethics statement

The experimental procedures were approved by the Experimental Animal Welfare and Ethical Committee of the Institute of Animal Science, Chinese Academy of Agriculture Sciences, Beijing, P. R. China (Ethics Approval Code: IAS2021-241). Written informed consent was obtained from the owners for the participation of their animals in this study.

## Author contributions

QG, GS, and YZ: designed the research. GS, CL, QG, and JD: conducted the experiments. QG and GS: analyzed the data. QG, RZ, and GS: wrote and revised the manuscript. LC, HZ, BW, CY, and YZ: provided the funding and supervision. All authors contributed to the article and approved the submitted version.

## Funding

This study was funded by the Tibet Science and Technology Projects (XZ-2019-NK-NS-003), the Fundamental Research Funds for the Chinese Academy of Agricultural Science (2022-YWF-ZX-03) in China, and Agricultural Science and Technology Innovation Program (CAAS-ZDRW202006-02 and ASTIPIAS07).

## Conflict of interest

The authors declare that the research was conducted in the absence of any commercial or financial relationships that could be construed as a potential conflict of interest.

## Publisher’s note

All claims expressed in this article are solely those of the authors and do not necessarily represent those of their affiliated organizations, or those of the publisher, the editors and the reviewers. Any product that may be evaluated in this article, or claim that may be made by its manufacturer, is not guaranteed or endorsed by the publisher.

## Footnotes

^1^ http://drive5.com/uparse/

^2^ http://rdp.cme.msu.edu/

^3^ www.i-sanger.com
