# Supplementary material for: Alterations in gut microbiota improve SCFA production and fiber utilization in Tibetan pigs fed alfalfa diet
Source: Front Microbiol. 2022 Oct 21;13:969524. doi: 10.3389/fmicb.2022.969524 (PMC9634421; doi:10.3389/fmicb.2022.969524)
Supplement: Supplementary file 1 [file Data_Sheet_1.docx]

***Supplementary Material***

**Alterations in gut microbiota improve SCFA production and fiber utilization in** **Tibetan pigs fed alfalfa diet**

Qingtao Gao^1,2 †^, Guangming Sun^1 †^, Jiujun Duan^2^, Chengzeng Luo^2,3^, Cidan-yangji^1^, Ruqing Zhong^2^, Liang Chen^2^, Yanbin Zhu^1 *^, Basang-wangdui^1^, and Hongfu Zhang^2^

^1^Institute of Animal Husbandry and Veterinary Medicine, Tibet Academy of Agriculture and Animal Husbandry Science, Lhasa, China

^2^The State Key Laboratory of Animal Nutrition, Institute of Animal Sciences, Chinese Academy of Agricultural Sciences, Beijing, China

^3^College of Animal Science, Xinjiang Agricultural University, Urumqi, China

***** Correspondence: zhuyanbin163@163.com, Yanbin Zhu

**^†^** Authors contributed equally to this article.


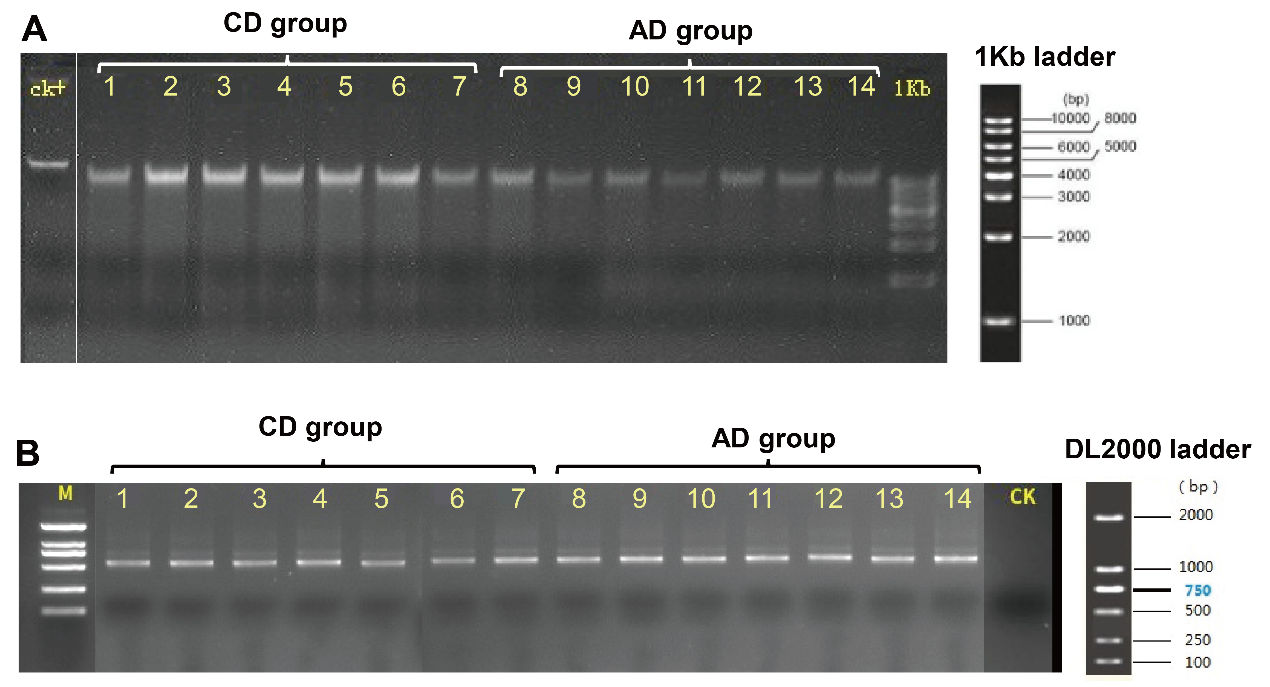
**Figure S1.** The agarose gel electrophoresis. The agarose gel picture of extracted DNA quality (A) and PCR product (B) for each sample of colonic digesta.


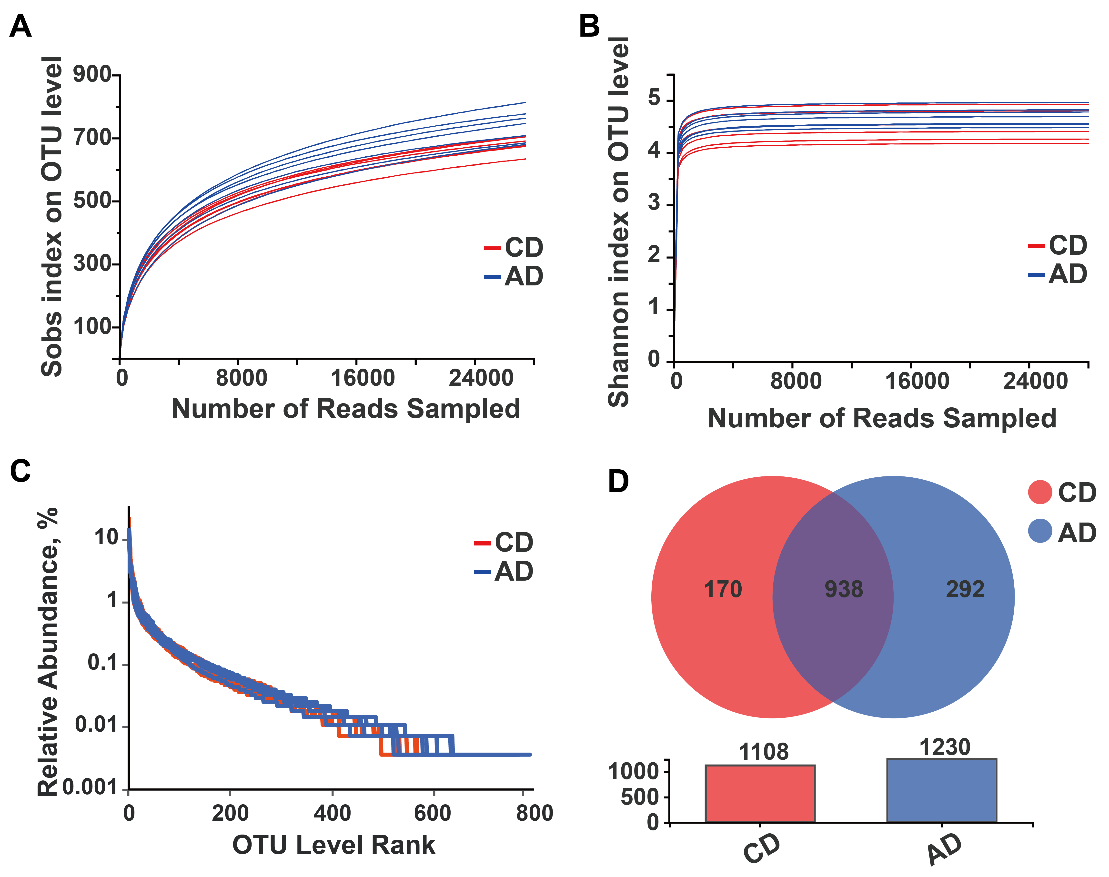
**Figure S2.** Evaluation of sample depth and Venn diagram. (A) Rarefaction curves of Sobs index; (B) Rarefaction curves of Shannon index; (C) Rank Abundance curves; (D) Venn diagram of OTUs. CD, control diet; AD, alfalfa diet.


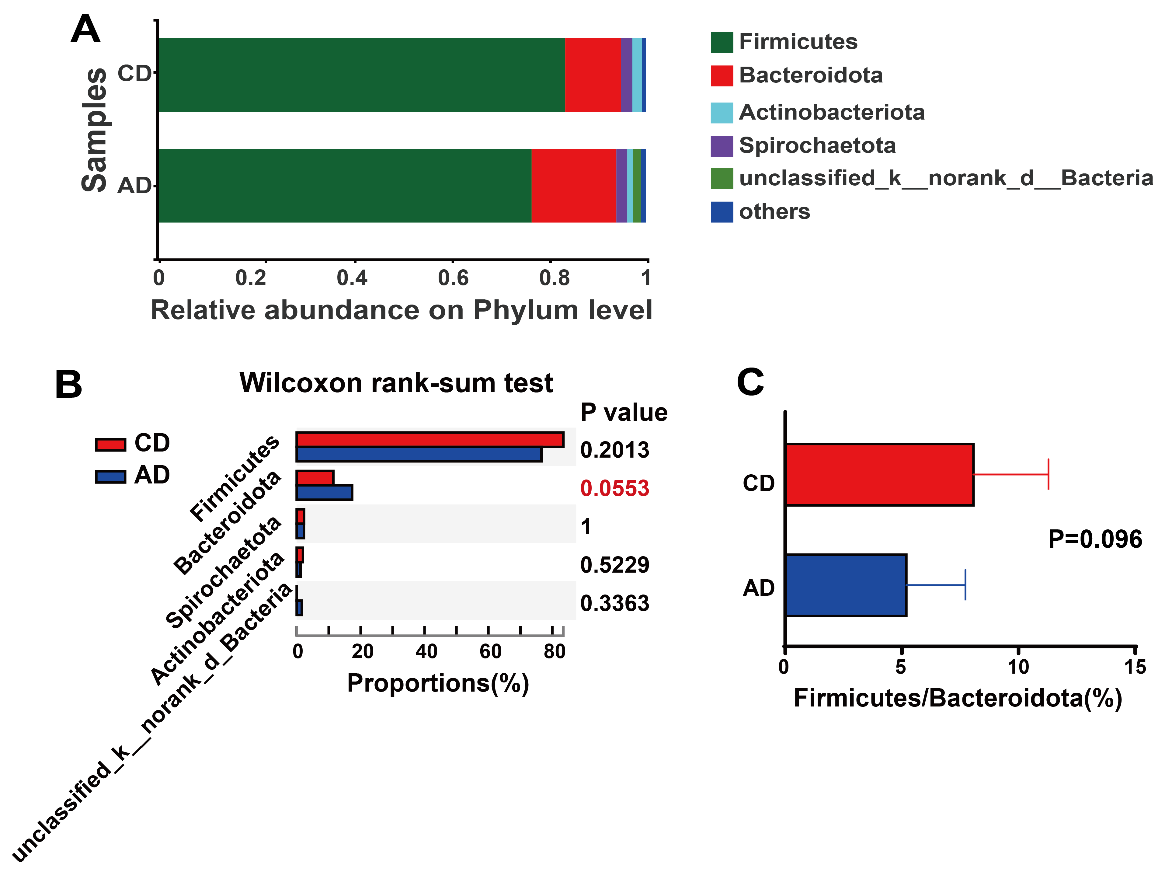
**Figure S3** Microbiota composition of colonic digesta and relative abundance (> 0.01) at phylum (A). Wilcoxon rank-sum test of microbes with a relative abundance of over than 1% (B) and ratio of Firmicutes to Bacteroidota (C).


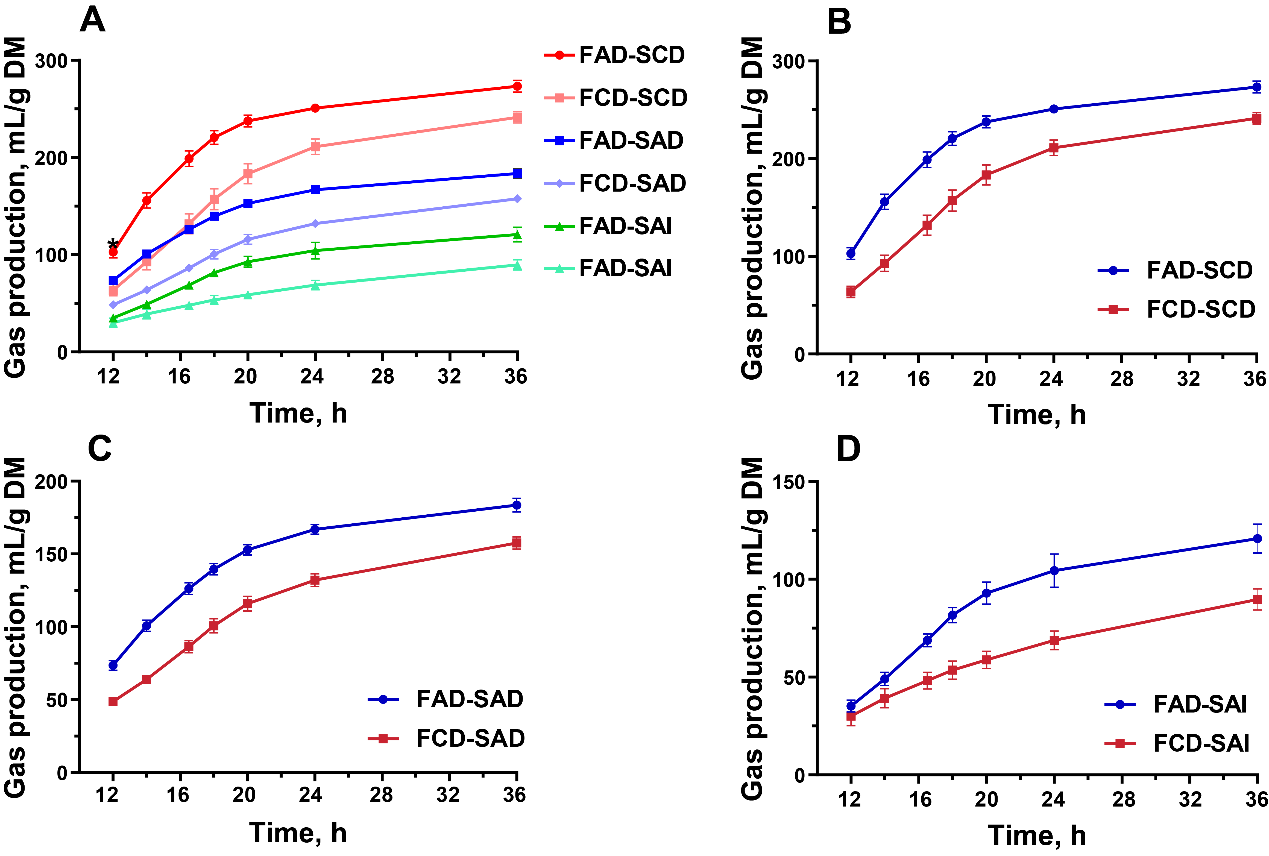
**Figure S4** Gas production curves for the in vitro batch fermentation. FAD, feces of pig fed AD diet; FCD, feces of pig fed CD diet; SCD, substrate of CD diet; SAD, substrate of AD diet; SAI, substrate of alfalfa ingredient.
